# Polyaniline/Carbon Nanotubes Composite Modified Anode via Graft Polymerization and Self-Assembling for Microbial Fuel Cells

**DOI:** 10.3390/polym10070759

**Published:** 2018-07-10

**Authors:** Wenguo Wu, Hao Niu, Dayun Yang, Shibin Wang, Nina Jiang, Jiefu Wang, Jia Lin, Chaoyi Hu

**Affiliations:** 1 College of Chemical Engineering, Huaqiao University, Xiamen 361021, China; 1400215014@hqu.edu.cn (H.N.); sbwang@hqu.edu.cn (S.W.); ninajiang@hqu.edu.cn (N.J.); jiefuwangxiamen@163.com (J.W.); linjia3456789@163.com (J.L.); 18559628110@163.com (C.H.); 2 Institute of Biomaterials and Tissue Engineering, Huaqiao University, Xiamen 361021, China; 3 Fujian Provincial Key Laboratory of Biochemical Technology, Xiamen 361021, China; 4 Fujian Key Laboratory for Translational Research in Cancer and Neurodegenerative Diseases, Institute for Translational Medicine, Fujian Medical University, Fuzhou 350108, China

**Keywords:** microbial fuel cell, carbon nanotubes, polyaniline, graft polymerization, self-assembling

## Abstract

Microbial fuel cells (MFCs) are promising devices for sustainable energy production, wastewater treatment and biosensors. Anode materials directly interact with electricigens and accept electrons between cells, playing an important role in determining the performance of MFCs. In this study, a novel carbon nanotubes (CNTs) and polyaniline (PANI) nanocomposite film modified Indium-tin oxide (ITO) anode was fabricated through graft polymerization of PANI after the modification of γ-aminopropyltriethoxysilane (APTES) on ITO substrate, which was followed by layer-by-layer (LBL) self-assembling of CNTs and PANI alternatively on its surface. (CNTs/PANI)_n_/APTES/ITO electrode with low charge transfer resistance showed better electrochemical behavior compared to the bare ITO electrode. Twelve layers of CNTs/PANI decorated ITO electrode with an optimal nanoporous network exhibited superior biocatalytic properties with a maximal current density of 6.98 µA/cm^2^, which is 26-fold higher than that of conventional ITO electrode in *Shewanella loihica* PV-4 bioelectrochemical system. MFCs with (CNTs/PANI)_12_/APTES/ITO as the anode harvested a maximum output power density of 34.51 mW/m^2^, which is 7.5-fold higher than that of the unmodified ITO electrode. These results demonstrate that (CNTs/PANI)_12/_APTES/ITO electrode has superior electrochemical and electrocatalytic properties compared to the bare ITO electrode, while the cellular toxicity of CNTs has an effect on the performance of MFC with (CNTs/PANI)_n_/APTES/ITO electrode.

## 1. Introduction

Microbial fuel cells (MFCs) are apparatus, which use electricigens as biocatalysts to harvest electrical energy from waste and biomass [1,2]. As a sustainable and environmentally friendly technology, MFCs are promising not only in sustainable energy production but also in wastewater treatment and biosensors [3,4,5,6]. Therefore, improving the performance of MFCs with high power density and efficiency has attracted significant interest in the past decade. Even though some impressive improvements have been made during recent years, the low power density still restricts the development and practical application of MFCs. The output power density of MFCs is affected by many factors, such as microorganisms, electrodes, configuration, operating conditions and so on. Among them, the property and surface structure of the anode materials that directly interact with electricigens and accept electrons from microorganisms play an important part in determining the performance of MFCs [7].

Graphite felt and carbon cloth are commonly used as suitable anodes in MFCs due to their chemical stability [8]. To increase the power output of MFCs, activated carbon and carbon brush with a larger surface area have been used as anodes in MFCs [9,10]. The anode materials as well as its surface structure and ability to interact with the bacteria are critical in determining MFC performance [11]. Carbon nanotubes are promising materials for designing or modifying electrodes in MFCs to improve the power density of MFCs, which have been widely used as electrochemical filters and catalytic membranes for degradation of organic pollutants [12,13,14] due to their large surface area, high conductivity and direct electron transfer characteristics [11,15]. Due to the high cost of CNTs, they are usually used to modify electrode materials that are not used in bulk. CNTs-modified anodes show a well-defined nanostructure and exhibit excellent electrochemical and catalytic properties. For example, polyethyleneimine/CNTs multilayers modified carbon paper created by layer-by-layer (LBL) assembly was found to decrease the internal resistance of MFCs and increase the power density by 20% [11]. CNT-coated graphite electrodes via electrophoretic deposition increase the initial cell attachment and biofilm proliferation, which results in higher current density [16]. In order to investigate the influence of different nanostructured electrodes on the performance of MFCs, vertically aligned CNTs, randomly aligned CNTs and spin-spray LBL CNTs based electrodes are prepared. Biofilms grown on them show different morphologies and thicknesses [17]. A CNT-textile anode is fabricated by coating CNTs on macroscale porous polyester fibers, which facilitates substrate transfer and internal bacterial colonization. The homogeneously coated CNTs promote active surface interaction with the microbial biofilm and facilitate electron transfer between the bacteria and the anode [18]. However, CNTs have cellular toxicity and could lead to proliferation inhibition and cell death [19]. Therefore, they are used in MFC after modification to reduce the cellular toxicity [20].

Several polymer materials were used to fabricate anodes in MFCs to improve anodic bioelectrocatalysis, such as osmium polymer and carbon nanofiber from polyacrylonitrile [21,22]. As a conductive polymer, polyaniline (PANI) has been widely used for surface modification of anodes in MFCs due to its facile processability, stability and biocompatibility [23,24]. The positively charged PANI in a neutral environment interacts with the negatively charged bacteria before promoting cell attachment and biofilm proliferation [25,26,27,28]. However, PANI possesses a small surface area and poor conductivity, which switches from an insulator to a conductor by protonic acid doping [27]. Therefore, we combined biocompatible PANI with conductive CNTs with a high surface area to reduce the cellular toxicity of CNTs and increase the conductivity of PANI to enhance the performance of anodes in MFCs. CNTs were deposited on PANI electropolymerized macroporous graphite felt (GF) by binder-free electrophoresis. The CNT/PANI/GF anode increased bacteria attachment on the electrode surface and the power density of MFCs [23]. Using the chemical vapor deposition technique, CNTs were grown on a carbon felt with the advantage of in-situ growth and graphene (GR), which was followed by modifying graphene (GR) and PANI on its surface. The performance of MFCs with GR/PANI-CNTs as anodes was improved and an optimum removal rate of 83% for chemical oxygen demand (COD) was obtained [29].

However, in the reported CNT/PANI/GF and GR/PANI-CNTs anodes, PANI was electropolymerized or physically adsorbed to the substrates. It is well-known that electropolymerization is usually used for in the synthesis and preparation of materials on a small scale, with the morphologies of materials being mainly determined by the reaction conditions [30]. Meanwhile, physical adhesion is unfavorable for the stability of the composite materials in the long term. Graft polymerization together with self-assembling technology can design and functionalize the substrate surfaces by the required polymers in a precise and homogenous manner [31,32]. This covalent binding of the grafted chains onto the surfaces of the materials avoids detachment of the chains and maintains the long-term chemical stability that is favorable for practical applications. The modification of γ-aminopropyltriethoxysilane (APTES) on the surface of TiO_2_ nanoparticles to allow for further graft polymerization of PANI was used for the preparation of PANI-TiO_2_ nanoparticles with good photocatalytic activity in the degradation of methyl orange [33] and arrays of PANI-APTES-TiO_2_ nanotubes with superior capacitance and electrochemical performance [30].

In this work, a self-assembled monolayer of APTES was formed on the surface of the ITO electrode and a dense PANI layer was further formed through oxidative graft polymerization of aniline monomer via covalent bonding. After this, CNTs with negatively charged functional groups (carboxyl) alternatively interacted with positively charged PANI through electrostatic attraction to form a homogeneous and stable composite multilayer film by the layer-by-layer (LBL) assembling method. The electrochemical and electricity generation performance of MFCs with (CNTs/PANI)_n_/APTES/ITO electrodes as anodes and *Shewanella loihica* PV-4 as electricigens in a single-chamber three-electrode electrochemical system and dual-chamber MFC were investigated.

## 2. Materials and Methods

### 2.1. Materials

Indium–tin oxide (ITO) conductive glasses were obtained from Hong Kong physical and chemical Co., Ltd, Beijing, China. MWCNTs (99.8%, 400 nm diameters) obtained from Times Nano Co., Ltd. (Chengdu, China) were purified and oxidized before use. Polyaniline (PANI) and γ-aminopropyltriethoxysilane (APTES) were purchased from Aldrich chemical Co., Ltd, Shanghai, China. The Dupont proton exchange membrane was purchased from Beijing honghaitian science and technology Co., Ltd. (Beijing, China). Other chemicals, including nitric acid, sulfuric acid, hydrogen peroxide, potassium ferricyanide, lactate sodium, sodium bicarbonate, ammonium chloride and sodium chloride, were purchased from Sinopharm Chemical Reagent Co., Ltd. (Beijing, China).

### 2.2. Modification of ITO Electrode via APTES

The ITO electrode was first sonicated in 4% of NaOH solution for 10 min to form hydroxyl groups on the surface. The pretreated ITO electrode was further immersed in APTES–anhydrous methylbenzene solution (1.1 g in 25 mL of methylbenzene) for 16 h with nitrogen, before being washed with methylbenzene, ethanol and dd-H_2_O to remove untreated APTES. The APTES modified ITO electrode was dried by nitrogen.

### 2.3. Fabrication of (CNTs/PANI)_n_/APTES/ITO Electrode

Firstly, the CNTs were sonicated in a mixture of 3:1 of H_2_SO_4_-HNO_3_ for 24 h, before being filtered and washed with ultrapure water. The CNTs functionalized with carboxylic acid groups were dispersed in dd-H_2_O by ultrasonication to obtain a homogeneous solution (1 mg/mL). Secondly, 100 mg of aniline was stirred with 100 mg of camphorsulfonic acid (HCSA) in 20 mL of chloroform until complete volatilization of chloroform. After this, the mixture was resuspended in dd-H_2_O (1 mg/mL) and sonicated for 1 h. Thirdly, the APTES coated ITO electrode mentioned above was immersed in the mixture solution for 30 min to chemically graft a PANI layer on the electrode surface. Finally, CNTs/PANI multilayer film was produced by alternately dipping the electrode into the negatively charged CNTs solution and positively charged PANI solution for 30 min each. Meanwhile, the electrode was washed with dd-H_2_O for 5 min after each coating step, before the electrode was dried in hot air. By repeating the procedures above, (CNTs/PANI)_n_/APTES/ITO electrode with different bilayers (*n* = 1, 3, 6, 8, 12 and 15) were obtained.

### 2.4. Bacteria Culture

*Shewanella loihica* PV-4 strain (ATCC BAA-1088) was aerobically cultured in Marine Broth (20 g/L) at 30 °C for 24 h. After centrifugation, the Marine Broth was replaced with defined media (NaHCO_3_ 2.5 g/L; CaCl_2_·2H_2_O 0.08 g/L; NH_4_Cl 1.0 g/L; MgCl_2_·6H_2_O 0.2 g/L; NaCl 10 g/L; 2-[4-(2-Hydroxyethyl)-1-piperazinyl]ethanesulfonic acid 7.2 g/L) [21] at 30 °C for 24 h with lactate sodium (10 mM) used as the substrate. All the cell suspension was centrifuged for 10 min, while the resultant cell suspension was washed with defined media three times prior to being used for electrochemical experiments and MFC tests.

### 2.5. Electrochemical Measurements

A single-chamber, three-electrode system was utilized for the electrochemical measurements. The CNTs/PANI multilayer film modified ITO electrode or bare ITO electrode (projected surface area: 3.14 cm^2^) was used as the working electrode and placed at the bottom of the cell. Furthermore, we used a platinum wire as the counter and an Ag/AgCl (saturated KCl) electrode as the reference. The cell was filled with 4 mL of defined media containing lactate sodium, before being deaerated by purging N_2_ gas for 30 min. After this, the bacteria with OD_600_ of 2.0 were injected into the cell at a constant poised potential of 0.2 V using a CHI 660D potentiostat (CH Instruments, Chenhua Co., Shanghai, China) at 30 °C with a pH of 7.8. All these experiments were performed in triplicate.

### 2.6. Scanning Electron Microscopy (SEM)

*S. loihica* PV-4 attached on the electrodes were imaged using a SEM (HITACHI, S-4800 and SU8010 UHR FE-SEM, Tokyo, Japan). Samples were fixed in 2.5% glutaraldehyde for 2 h, rinsed thrice using phosphate buffer (pH of 7.0, 50 mM), dehydrated by alcoholic series (60%, 70%, 80%, 90%, 95% and 100%) and finally air-dried. Samples were subjected to SEM observations.

### 2.7. Polarization Curve Measurement

A dual-chamber MFC with two equal rectangular cells of 14 mL in volume was constructed. The CNTs/PANI multilayer film modified ITO electrode or bare ITO electrode was employed as the anode. The plain carbon cloth was used as the cathode. A Nafion PEM was sandwiched between the two chambers. The anode, cathode and PEM all had the same circular area of 7 cm^2^. The cathodic compartment was fed with 10 mM ferricyanide in PBS solution (pH of 7.4, 100 mM). The anodic compartment was fed with *S. loihica* PV-4 inoculated defined media containing lactate sodium. The power output and polarization curves were obtained by varying the external resistor from 50–10,000 Ω when the current output reached a steady state. The voltage was recorded by a multi-channel data acquisition board (NI USB-6008, Beijing huatai Orient technology Co., Ltd., Beijing, China). All these results were repeated three times.

## 3. Results and Discussion

### 3.1. Characterization of (CNTs/PANI)n/APTES/ITO Electrode

**Figure 1:**
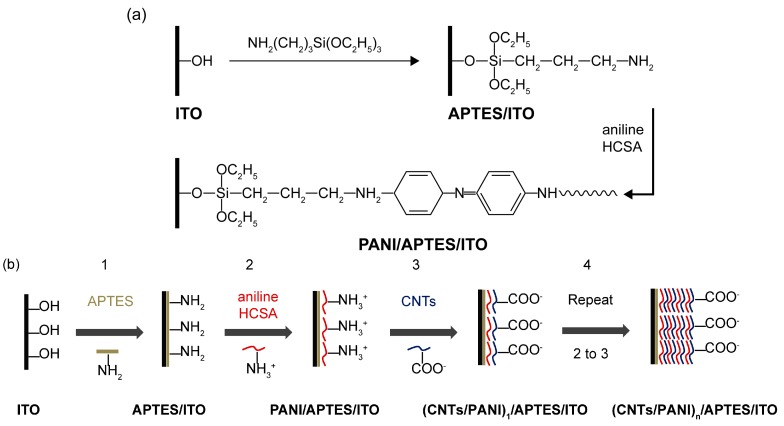
Mechanism of modification of PANI on ITO electrode via APTES (**a**) and schematic illustration of the fabrication process of (CNTs/PANI)_n_/APTES/ITO electrode (**b**).

The mechanism of deposition of PANI via graft polymerization after the modification of APTES on the alkalized ITO electrode and the fabrication process of (CNTs/PANI)_n_/APTES/ITO electrode are shown in Figure 1. The ending –NH_2_ of APTES serves as an initiated site for polymerization, while the aniline monomer undergoes oxidized polymerization with the addition of HCSA. Finally, PANI is successfully grafted on the surface of ITO electrode [30]. Since PANI is in the protonated state, there are electrostatic interactions between carboxyl functionalized CNTs and amino of PANI as well as π–stacking between aromatic rings of PANI and CNTs [34].

**Figure 2:**
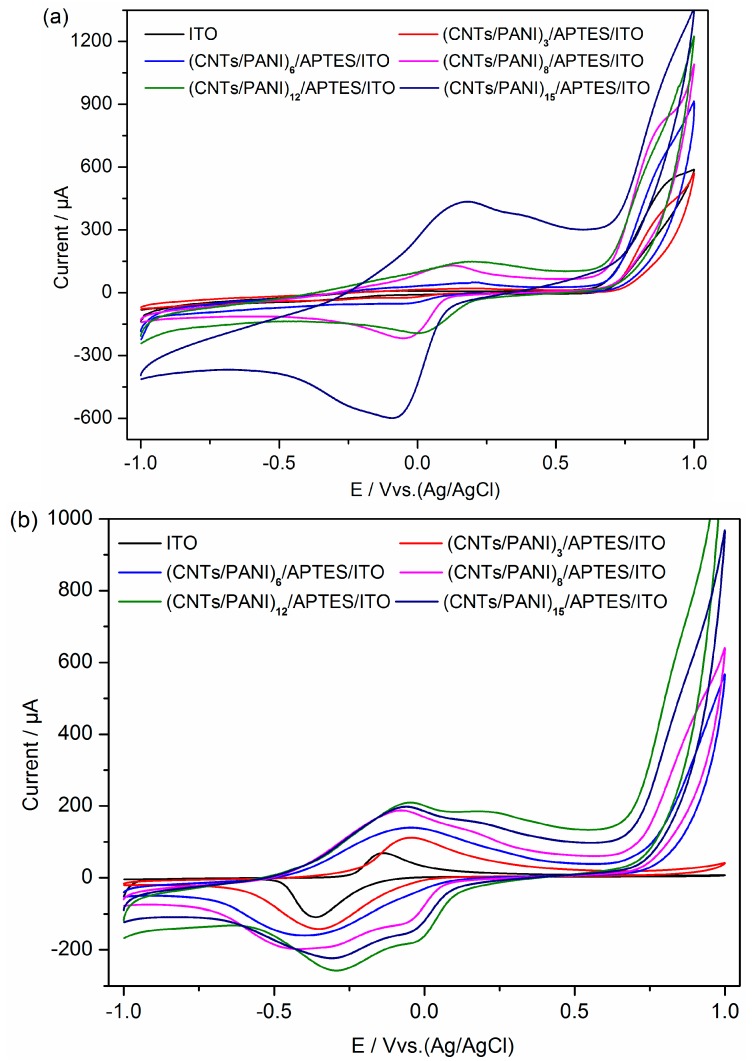
The cyclic voltammetry (CV) of ITO electrode and (CNTs/PANI)_n_/APTES/ITO electrode before inoculation (**a**) and at the end of batch tests (**b**) in bioelectrochemical cell with a scan rate of 10 mV/s. *n* = 3, 6, 8, 12 and 15.

The cyclic voltammetry (CV) profiles of plain ITO electrode and (CNTs/PANI)_n_/APTES/ITO electrode before inoculation are shown in Figure 2a. ITO electrode displays no obvious redox waves, while a couple of well-formed redox peaks at the potentials ranging between −0.07 and 0.18 V is evident on (CNTs/PANI)_n_/APTES/ITO electrode. The peak current increases with an increase in layer number. It has been reported that the characteristic redox peaks originate from the redox transition of PANI between the leucoemeraldine and the polaronicemeraldine form [23,28,35]. The results confirm the successful deposition of CNTs/PANI composite film on ITO electrode.

The electrochemical performance of (CNTs/PANI)_n_/APTES/ITO electrode with *S. loihica* PV-4 is significantly enhanced compared to that of the bare ITO electrode (Figure 2b). A pair of redox peaks at the potential of −0.28 and −0.04 V is observed, which originated from the outer-membrane protein of *c*-Cyts directly transferring electrons from *S. loihica* PV-4 cells to the electrode surface [24]. The peaks at the bare ITO anode at around −0.1 and −0.4 V were also attributed to the outer membrane of *c*-Cyts. As reported previously, the midpoint potential (*E*_m_) of *c*-Cyts is dependent on their redox state. The negative shift of *E*_m_ reflected the oxidization of *c*-Cyts, which stabilized the redox states of *c*-Cyts in a feedback manner [36,37]. The CV curves become well-defined quasi-reversible, which is attributed to the increasing surface area of electrode modified with CNTs/PANI composite film. Meanwhile, the redox current is obviously increased with an increase in multilayer number and further decreased at 15. There is an appearance of new redox peaks with an anodic potential of about 0.25 V and a cathodic potential of about −0.04 V as the bilayer number increases from 8 to 15. It was reported that the redox peaks at the potential of −0.17 and 0.67 V are the characteristic redox peaks of PANI, while the anodic peak at about 0.25 V reflects the transition from leucoemeraldine to protonated emeraldine, which is the most conductive form of PANI [24,34]. The electrochemical behavior of (CNTs/PANI)_n_/APTES/ITO electrode is greatly enhanced compared with the plain ITO electrode, with (CNTs/PANI)_12_/APTES/ITO electrode exhibiting superior catalytic properties in electron transfer with the highest redox currents of 209.90 and −257.54 µA. However, the peak currents in CVs at the end of batch tests are lower than those before inoculation. It was reported that CNTs have antimicrobial activity and cellular toxicity [19]. These results indicated that the dead cells or cells with low activity on the electrode surface could reduce the active sites of the electrode and lead to the lower redox peak currents of PANI.

### 3.2. Chronoamperometric Results

**Figure 3:**
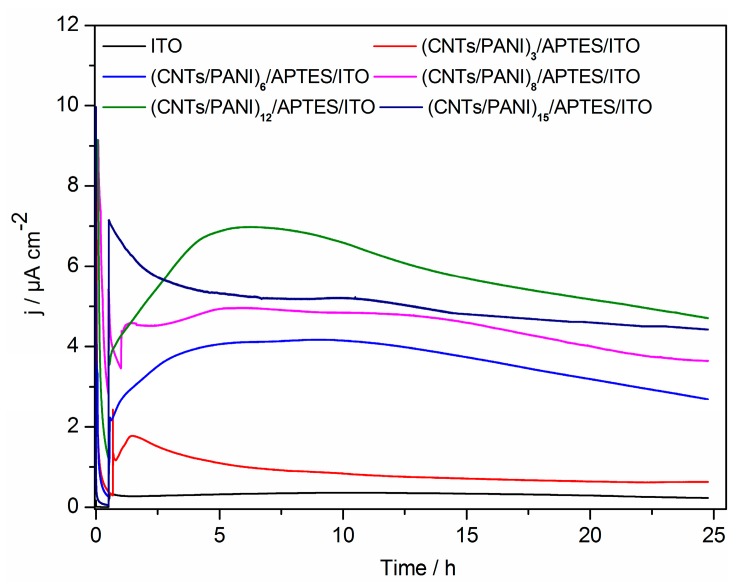
Current versus time measurement of current generation by *S. loihica* PV-4 cells on ITO electrode and (CNTs/PANI)_n_/APTES/ITO electrode poised at 0.2 V. *n* = 3, 6, 8, 12, 15.

The current density generated on (CNTs/PANI)_n_/APTES/ITO electrode and bare ITO electrode by *S. loihica* PV-4 cells at an applied potential of 0.2 V in the bioelectrochemical system is shown in Figure 3. There is an instant current with the addition of bacterial cells at 0.5 h. The oxidative current increases simultaneously to a peak current, before gradually decreasing to a gentle current. The oxidative current is ascribed to the interactions between bacterial cells and electrode surface, while the consumption of substrate results into an decrease in current. The current density is significantly improved when using (CNTs/PANI)_n_/APTES/ITO electrode as compared to that obtained when using the plain ITO electrode. This increases with increasing bilayer number from 0 to 12, while the current density is decreased at the number of 15. (CNTs/PANI)_12_/APTES/ITO electrode displays outstanding electrochemical performance with the maximal current density of 7.03 ± 1.6 µA/cm^2^, which is 26 times higher than that on the plain ITO electrode. This result is also higher than the previously reported 3.80 µA/cm^2^ on nanograss array boron doped diamond electrode (BDD) [37,38], 2.93 µA/cm^2^ on (Au/PAH)_6_/BDD electrode [39] and ~1.3 µA/cm^2^ on (PAH/PSS)_4_/(PAH/Fe_2_O_3_)_2_/PAH/ITO electrode [40]. Remarkably, the current density is also higher than that on the superhydrophilic ITO electrodes with or without the addition of riboflavin as a mediator [30]. This result indicates that CNTs/PANI conductive films with different multilayer numbers are successfully modified on the electrode, which greatly increases the electrode surface area and reaction activity sites in favor of the bacteria catalytic oxidation of the substrate [20]. Furthermore, the modification of PANI with positive charges increases the hydrophilicity of electrode surface and promotes electrostatic adhesion of bacteria with negative charges on the outer membrane [23,41]. In addition, the bacteria with negative function groups outside could dope in the polyaniline to keep the form of emeraldine-salt and retain the conductivity of PANI [42]. Although PANI with positive charges was modified on the surface of (CNTs/PANI)_n_/APTES/ITO electrode, the prepared electrode exhibited the most negatively charged carboxyl groups of CNTs at its outer surface. These negative charges could compete with the positively charged amino groups of PANI to repulse the bacteria with negative charge surfaces. Moreover, it was reported that CNTs have antimicrobial activity and cellular toxicity. An independent experiment of the repeated addition of sodium lactate per 25 h for 3 batches did not exhibit an increase in current and demonstrated the toxicity of CNTs in inhibiting the growth of bacteria.

### 3.3. SEM Results

**Figure 4:**
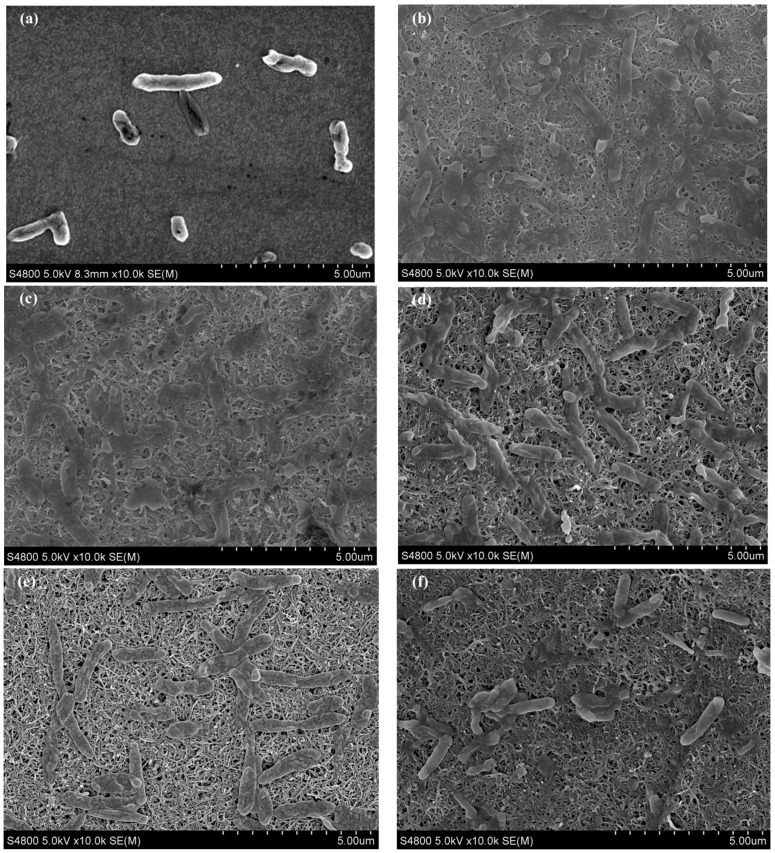
SEM images of *S. loihica* PV-4 cells on the surface of ITO electrode (**a**) and (CNTs/PANI)_n_/APTES/ITO electrode after 25 h of electrochemical culture (**b**: 3; **c**: 6; **d**: 8; **e**: 12; **f**: 15).

The SEM images in Figure 4 reveal that the multilayer CNTs/PANI deposited films are well formed on the surface of ITO electrodes and *S. loihica* PV-4 cells are firmly attached to the electrode surface. There are sparse bacterial cells in the shortened rod shape on the surface of ITO electrode (Figure 4a). Cell attachment on electrode surface is significantly increased after the modification of CNTs/PANI composite film. It is attributed to the high conductivity and effective surface area of CNTs as well as the interactions between the positively charged PANI and negatively charged bacterial cells [23,41]. This increase in cell adhesion should be one reason for the high current density and enhanced electrochemical behavior on (CNTs/PANI)_n_/APTES/ITO electrode. There is no significant difference in cell attachments on CNTs/PANI film deposited electrodes between the bilayer numbers of 3, 6 and 8, while cell adhesion is apparently decreased as the layer number increases to 12 and 15. Interestingly, several cells in the elongated rod shape with the length of about 3.5 µm are observed on (CNTs/PANI)_12_/APTES/ITO electrode (Figure 4e), which exhibits superior bioelectrochemical behavior. The inhibition of cell division by toxic CNTs could be the reason for the cell elongation [43]. The elongated cells could cover a large electrode surface area in a better way and resulted in high current density [44]. The increasing amount of CNTs on (CNTs/PANI)_15_/APTES/ITO electrode may cause the death of cells or cells with low activity on the electrode surface (Figure 4f), which could reduce the active sites of electrode and result in low performance of (CNTs/PANI)_15_/APTES/ITO electrode. Therefore, CNTs/PANI composite film modified ITO electrode with 12 bilayers not only achieves high conductivity and large surface area but also harvests the optimal surface morphology for adhesion of cells in an elongated shape that is favorable for substrate consumption and electron transfer.

### 3.4. Electrochemical Impedance Spectroscopy (EIS) Results

**Figure 5:**
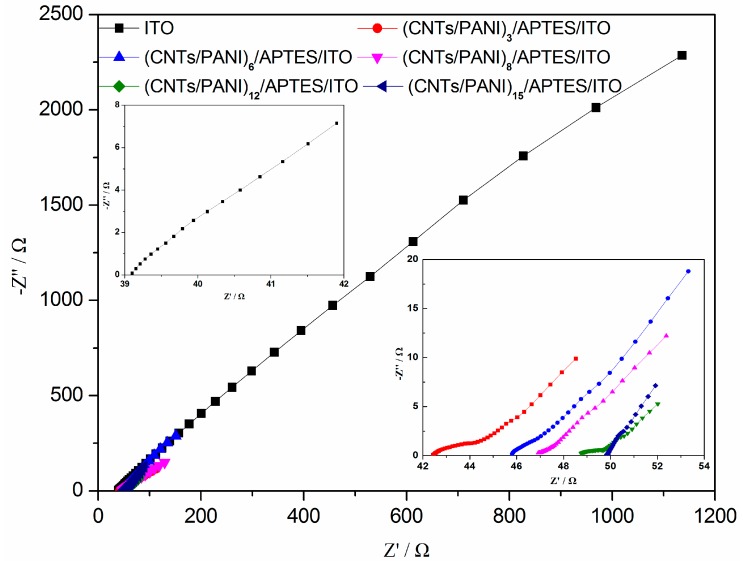
Nyquist plots on ITO electrode and (CNTs/PANI)_n_/APTES/ITO after 25 h of chronoamperometry (0.1–100 kHz at open-circuit potential and with a perturbation signal of 10 mV). The inset is the higher magnification of the high frequencies part. *n* = 3, 6, 8, 12 and 15.

The EIS Nyquist plot is carried out to study the electrochemical activity of CNTs and PANI composite film deposited electrodes. The limiting factors of a MFC are the representation of complex resistances, such as activation resistance, ohmic resistance and concentration resistance [45]. In this three-electrode bioelectrochemical system, the ohmic resistance (*R*s) stands for the resistance of electrolyte, electrode materials and modification layer, while the charge transfer resistance (*R*ct) represents the resistance of the electrochemical reaction on the electrode [46]. A smaller *R*s value is observed on the bare ITO electrode and the *R*s value increases with an increase in layer number. It indicates that the modification of CNTs/PANI film on ITO electrode increases the connection resistance between the modification layer and the ITO substrate. The *R*ct at an electrode–electrolyte interface is usually indicated by the diameter of semicircle in the high-frequency area, which is followed by the straight line at lower frequencies [24]. The result in Figure 5 shows that *R*ct is reduced with an increase in deposition layer from 0 to 12, while this is further increased with increasing CNTs/PANI modified layer. It suggests that the catalytic performance of electrodes can be significantly improved by incorporating a porous structure in the deposited CNTs/PANI layer, which increases the availability of electrochemically active sites and enhances the electron transfer rate [47,48]. (CNTs/PANI)_12_/APTES/ITO electrode with distinguished electrochemical performance suggests that it possesses a good nanostructured network for fast electron transfer on its surface.

### 3.5. Polarization Curves

**Figure 6:**
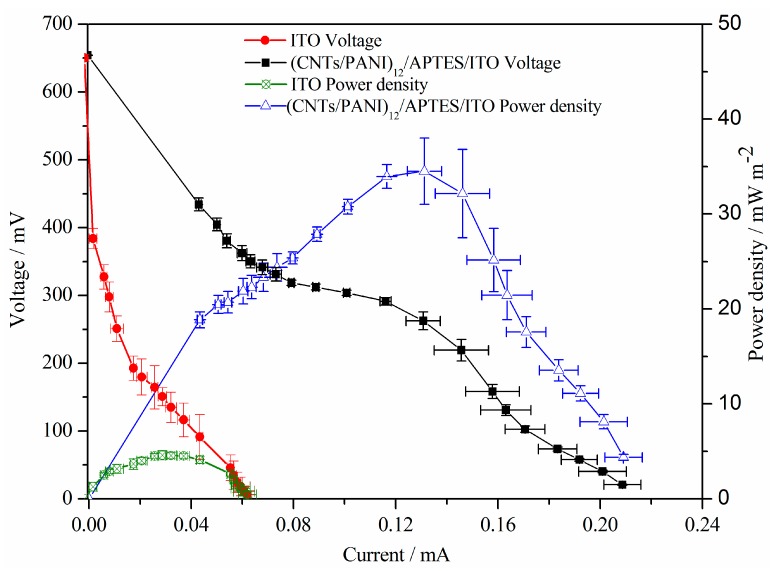
Power density output and polarization curves of MFCs with (CNTs/PANI)_12_/APTES/ITO anode and ITO anode.

To estimate the electricity generation performance of CNTs and PANI composite film modified ITO electrode in determining the potential of MFCs in practical applications, (CNTs/PANI)_12_/APTES/ITO electrode with excellent biocatalytic and electrochemical properties is chosen as the anode for further evaluation of the power density output and polarization curves of MFC. As shown in Figure 6, MFCs with 12 layers of CNTs/PANI film deposited ITO electrode deliver significantly higher power density compared to that of MFCs with a plain ITO electrode. The maximum power density of MFC with (CNTs/PANI)_12_/APTES/ITO electrode is 34.51 ± 3.49 mW/m^2^, which is 7.5 times higher than that obtained on the bare ITO electrode. The power density reaches its maximum when the external resistance equals the internal resistance of the MFC. The lower internal resistance of MFC with (CNTs/PANI)_12_/APTES/ITO anode indicates that the CNTs/PANI anodic modification accelerates the interfacial electron transfer between the bacteria and the electrode surface [46]. However, the maximum power density value from (CNTs/PANI)_12_/APTES/ITO anode MFC is lower than that of CNT/PANI paste anode MFC with *Escherichia coli* as the microbial catalyst (42 mW/m^2^) and ~7.5 times lower than that of CNT-125/PANI/GF anode MFC with *Shewanella putrefaciens* as an exoelectrogen (257 mW/m^2^) [20,23]. It was reported that the amount of CNTs in PANI/CNT composites affected the electrocatalytic behavior of the composite anode in MFCs [20]. Therefore, the optimized amount of CNTs in (CNTs/PANI)_12_/APTES/ITO anode should be further investigated to enhance the power density of MFC.

The charge-transfer resistance of MFCs, reflecting the electron transfer efficiency of the anode, can be determined from the slope of the second part of the polarization curves [35,49]. As shown in Figure 6, MFCs with CNTs/PANI film deposited ITO anode and plain ITO anode display almost the same open-circuit voltage of about +0.65 V due to the homogeneous electrolyte in MFCs [50]. As estimated from the polarization curves, the charge-transfer resistance of MFC with (CNTs/PANI)_12_/APTES/ITO anode is about 3.3 KΩ, which is about 3.7 times lower than that of MFC with the ITO anode. This result suggests that (CNTs/PANI)_12_/APTES/ITO anode has much faster electron transfer rate than ITO anode, which contributes to the improvement in power generation.

## 4. Conclusions

(CNTs/PANI)_n_/APTES/ITO electrodes are successfully obtained by graft polymerization of PANI via APTES, which is followed by self-assembling of CNTs and PANI alternatively on its surface. Blank CV results showed that the characteristic redox peaks of PANI were exhibited on CNTs/PANI film deposited electrode without bacteria. After the addition of cells, the peak currents of two pairs of redox peaks were increased from 0 to 12, before subsequently decreasing. Chronoamperometry and EIS analyses also showed similar results as the current density increased before decreasing at the layer number of 15, which corresponds to the charge transfer resistance first decreasing before increasing. SEM results showed that the cells in the elongated rod shape were attached on (CNTs/PANI)_12_/APTES/ITO electrode with a well-formed nanoporous structure. The power density of MFC with (CNTs/PANI)_12_/APTES/ITO anode reached 34.51 mW/m^2^ which is 7.5 times higher than that of the unmodified ITO electrode. The experimental results demonstrate that (CNTs/PANI)_12_/APTES/ITO electrode has superior electrochemical and electrocatalytic properties compared to the bare ITO electrode and that cellular toxicity of CNTs has an effect on the performance of MFC with (CNTs/PANI)_n_/APTES/ITO electrode. The optimized amount of CNTs in (CNTs/PANI)_12_/APTES/ITO electrode should be further investigated to enhance the power density and stability of long-term operation of MFCs.
